# Frailty Components as Moderators of the Subjective Cognitive Decline–Depressive Symptoms Association: A Sex-Stratified Population-Based Study

**DOI:** 10.3390/healthcare14162495

**Published:** 2026-08-11

**Authors:** Eun Sook Lee, Nam Joo Je, Yeong-Mi Seo

**Affiliations:** 1 Sustainable Health Research Institute, College of Nursing, Gyeongsang National University, Jinju-si 52727, Republic of Korea; eslee5335@gnu.ac.kr; 2 Department of Nursing, College of Nursing, Changshin University, Changwon-si 51352, Republic of Korea; jnj4757@cs.ac.kr

**Keywords:** cognitive dysfunction, depressive symptoms, frailty, sex, aged

## Abstract

**Highlights:**

**What are the main findings?**

- Subjective cognitive decline (SCD) was significantly associated with higher levels of depressive symptoms in men and women.
- Different individual frailty components were identified as significant moderators of the association between SCD and depressive symptoms in men and women.

**What are the implications of the main findings?**

- Assessment of depressive symptoms in older adults with SCD may be enhanced by considering specific frailty components rather than overall frailty status alone.
- The findings highlight the importance of examining individual frailty components in future research using sex-aware approaches to better understand cognitive and emotional health in older adults.

**Abstract:**

**Background/Objective:** Subjective cognitive decline (SCD) and depressive symptoms are among the most prevalent mental health concerns in older populations, and SCD has been consistently associated with depressive symptoms. However, whether individual Fried Frailty Phenotype (FFP) components modify the association between SCD and depressive symptoms, and whether these moderating patterns differ across sex, has yet to be empirically examined. This study aimed to examine whether individual FFP components moderate the association between SCD and depressive symptoms in older adults, using sex-stratified analyses. **Methods:** Using data from the Korea National Health and Nutrition Examination Survey, complex sample general linear models were applied to 1404 adults aged 65 years and older. Five FFP components were tested as moderators separately for men and women. **Results:** SCD was positively associated with depressive symptoms in both sexes. Unintentional weight loss (B = 0.43, *p* = 0.009) and exhaustion (B = 0.18, *p* = 0.037) were significant moderators in men. Among women, muscle weakness (B = −0.26, *p* = 0.002), slowness (B = 0.18, *p* = 0.020), and unintentional weight loss (B = −0.34, *p* = 0.022) were identified as significant moderators. Additionally, a formal interaction test indicated a statistically significant sex difference only for unintentional weight loss. **Conclusions:** The findings underscore the value of examining frailty at the individual-component level, rather than as a single construct, offering a more nuanced understanding of the association between SCD and depressive symptoms through sex-stratified analyses.

## 1. Introduction

According to the United Nations Department of Economic and Social Affairs (UN DESA), adults aged 65 years and older accounted for 10.3% of the global population in 2024, a proportion projected to reach 20.7% by 2074 [1]. Particularly, South Korea entered a super-aged society in 2025 as its older population surpassed 20%, and projections indicate that this proportion will reach approximately 46.4% by 2070, making it the most aged country globally [2]. This rapid demographic shift has intensified health challenges such as cognitive decline, depressive symptoms, and frailty, resulting in not only a decline in individuals’ quality of life but also socioeconomic losses [3,4].

To alleviate these socioeconomic burdens, scholarly attention has increasingly focused on subjective cognitive decline (SCD), a pre-clinical stage that precedes formal diagnosis. SCD refers to a self-perceived deterioration in cognitive function that occurs in the absence of objectively measurable deficits [5]. According to prior studies, SCD is regarded as an early warning sign that significantly increases the risk of progression to mild cognitive impairment (MCI) or dementia, with individuals with SCD showing more than double the risk of MCI or dementia compared to those without SCD [6,7].

SCD is closely associated with not only cognitive problems but also mental health, and individuals with SCD have been reported to be at increased risk of depressive symptoms [8,9,10]. Furthermore, longitudinal studies have suggested that SCD may precede the onset of depressive symptoms, and that SCD may function as both a precursor and significant predictor of depressive symptoms [10]. However, not all individuals with SCD experience depressive symptoms, and this association may vary depending on individual levels of vulnerability.

Frailty—a key concept in explaining individual vulnerability—refers to a state of decreased resistance to stressors due to reduced physiological reserves [11]. Fried et al. defined frailty using the Fried Frailty Phenotype (FFP), which has five components: weight loss, exhaustion, low physical activity, slowness, and muscle weakness [11]. Previous research has consistently associated frailty with both cognitive decline and depressive symptoms in older adults [12,13,14,15]. Furthermore, Nascimento et al. reported that frailty may reduce the ability to cope with cognitive changes, thereby moderating the relationship between cognitive decline and depressive symptoms [13]. They found that even at similar levels of cognitive decline, higher frailty was associated with a stronger relationship with depressive symptoms. However, these studies primarily focused on objectively measured cognitive decline [12,13]. As SCD is considered a prodromal stage of objective cognitive impairment [6,7], it is necessary to examine whether frailty has a moderating effect on the association between SCD and depressive symptoms.

Although each component of the FFP reflects physical functional decline, the magnitude of its association with cognitive and emotional health varies across individual items [16,17]. For example, exhaustion is closely associated with reduced concentration and working memory, while slowness is closely linked to executive function and information processing speed [16]. Additionally, weight loss, exhaustion, and reduced gait speed have shown significant associations with depressive symptoms [17]. Given these differential associations, it is plausible that individual FFP components may serve as distinct moderating factors in the relationship between SCD and depressive symptoms. However, rather than examining the individual components of frailty, most prior studies have conceptualized it as a unified construct or limited their analyses to independent associations between individual components and cognition or depressive symptoms [12,13,14,15,16,17]. In addition, studies on the moderating role of frailty in the relationship between cognitive function and depressive symptoms are limited [13]. Consequently, research investigating the moderating effects of individual FFP components on the relationship between SCD and depressive symptoms also remains scarce. Therefore, more precise analyses of the independent roles of individual FFP components are warranted. Such an approach may enhance the current understanding of the specific physical vulnerabilities related to cognitive and emotional health while contributing to the development of more targeted intervention strategies.

In analyzing the moderating effects of the FFP components on the relationship between SCD and depressive symptoms, it is essential to consider sex. The prevalence of FFP components varies by sex. Men exhibit the highest prevalence of muscle weakness, whereas women demonstrate a higher prevalence of low physical activity and exhaustion compared to men [18]. Furthermore, the associations between frailty components and depressive symptoms also differ by sex. In men, muscle weakness and slow gait speed were reported to be significantly associated with depressive symptoms, whereas in women, low physical activity is strongly associated with depressive symptoms [19]. These findings suggest sex differences in the associations between frailty components and emotional health. Therefore, it is plausible that the moderating effects of individual FFP components on the association between SCD and depressive symptoms may also differ between men and women. Nevertheless, studies that explicitly compare the moderating effects of FFP components by sex remain scarce, and research focusing on older adults in Korea is particularly limited.

Therefore, this study utilized nationally representative data from the Korea National Health and Nutrition Examination Survey (KNHANES) to examine whether individual FFP components moderate the association between SCD and depressive symptoms. Recognizing that frailty manifests differently in men and women, this study aimed to examine the moderating effects of FFP components within each group through sex-stratified analyses and to compare the patterns between men and women. This component-level, sex-stratified approach may offer a more nuanced understanding of the relationship between SCD and depressive symptoms than prior studies that treated frailty as a single construct, with potential relevance for sex-aware approaches to ensuring cognitive and emotional health among older adults. We hypothesized that: (1) SCD is significantly associated with depressive symptoms in Korean community-dwelling older adults. (2) Individual FFP components, including weight loss, exhaustion, low physical activity, slowness, and muscle weakness, moderate the association between SCD and depressive symptoms. (3) The patterns of significant moderators identified in sex-stratified analyses may differ between men and women.

## 2. Materials and Methods

### 2.1. Study Design

This study employed a cross-sectional descriptive design using secondary data to examine whether FFP components moderate the relationship between SCD and depressive symptoms.

### 2.2. Participants and Data Collection

This study was conducted as a secondary analysis using data from the 9th cycle, 2nd year (2023) of the KNHANES. KNHANES is a population-based cross-sectional survey covering all 17 metropolitan and provincial regions of South Korea, employing a stratified two-stage cluster sampling design based on national population and housing census data. The survey was administered directly by trained interviewers and medical personnel following standardized procedures, and included household member identification, health questionnaires, physical examinations, and nutritional assessments.

In this study, key variables were derived from relevant items in the health questionnaire and physical examination components. The analysis was restricted to 1836 adults aged 65 years and older among the total 6929 respondents. Missing data were identified in SCD, depressive symptoms, and the following FFP components: weight loss (*n* = 1), slowness (*n* = 92), low physical activity (*n* = 228), exhaustion (*n* = 97), and muscle weakness (*n* = 246). Respondents with missing data on any of these variables were excluded (*n* = 432), as some participants had missing data on multiple variables simultaneously. Muscle weakness was assessed using grip strength test results; participants who were unable to complete the test due to physical reasons, including a recent history of hand or wrist surgery or the experience of pain, aching, or stiffness in the hands, were excluded based on pre-examination visual inspection and questionnaire criteria [20]. A total of 1404 participants with no missing data on key variables were included in the final analysis.

### 2.3. Measures

The measurement items used in this study were derived from the KNHANES, a nationally representative health survey in South Korea. KNHANES produces health statistics with national-level representativeness and data reliability through a standardized survey system and rigorous quality control procedures [21]. In this study, SCD, depressive symptoms, and select frailty components were measured using individual items from the Health-Related Quality of Life Instrument with 8 Items (HINT-8). The HINT-8 is an instrument designed to measure eight independent constructs (stair climbing, pain, energy, work, depressive symptoms, memory, sleep, and happiness) using a single item for each. The instrument has demonstrated acceptable psychometric properties [22].

### 2.4. SCD

SCD was assessed using the memory item of the HINT-8. The memory item measures the degree of subjective memory difficulty through a self-report format, “I had difficulty remembering things,” on a 4-point Likert scale ranging from 1 (no problem) to 4 (the problem is very severe). The reliability of the item was reported as Cohen’s kappa = 0.416, weighted kappa = 0.464, and agreement rate = 70% [23]. A single self-reported memory item had been used as an SCD indicator in previous studies as a practical alternative in large-scale epidemiological surveys [14,24]. In this study, the memory item score was used as a continuous variable, with higher scores indicating greater SCD. Continuous measurement of SCD has been reported to better capture the association between SCD and cognitive function compared to dichotomous measurement [25].

### 2.5. Depressive Symptoms

Depressive symptoms were assessed using the depression item of the HINT-8. This item measures recently experienced depressive feelings on a 4-point Likert scale ranging from 1 (not depressed at all) to 4 (always depressed), with higher scores indicating greater levels of depressive symptoms. This item reflects self-reported depressive symptoms rather than a clinical diagnosis of depressive disorder. The item’s reliability was reported as Cohen’s kappa = 0.436, weighted kappa = 0.513, and agreement rate = 68%, indicating moderate reliability [23]. Although this is a single-item measure, prior studies have utilized it as an indicator of depressive status [14,24]. In this study, the depressive symptoms item score was used as a continuous variable to reflect the continuum of depression levels and to improve the precision of interaction estimation [26].

### 2.6. Frailty

We utilized a modified FFP adapted from the criteria proposed by Fried et al. [11] to align with the characteristics of the Korean older adult population and the measurement structure of the KNHANES data. Frailty was assessed across five components: unintentional weight loss, exhaustion, low physical activity, slowness, and muscle weakness. First, unintentional weight loss was defined as a body weight reduction of 3 kg or more over the past year without deliberate effort, based on established epidemiological survey criteria [20,27]. Second, exhaustion was assessed using the vitality item of the HINT-8. In large-scale epidemiological surveys, self-reported items asking about recent energy or vitality levels have been used as practical proxy indicators of exhaustion [14,27]. The vitality item assesses energy status, and based on prior research [28], responses of “I had energy sometimes (3 points)” or “I had no energy at all (4 points)” were classified as exhaustion. Third, muscle weakness was assessed using a digital hand dynamometer (TKK 5401, Takei Scientific Instruments Co., Ltd., Niigata, Japan). Three measurements were obtained for each hand, and the maximum value was retained. Cases with grip strength below 28 kg for men and below 18 kg for women were classified as muscle weakness, based on the AWGS 2019 criteria [29]. Grip strength is a representative indicator of physical function and has been widely utilized as a frailty indicator in previous studies [12,17]. Fourth, slowness was assessed using the stair climbing item of the HINT-8, which measures the degree of difficulty in climbing stairs. Because KNHANES does not include an objective gait speed assessment, the stair climbing item was used as a proxy measure for the slowness component of the original FFP. Difficulty in stair climbing has been reported to be closely associated with gait speed [30]. Furthermore, previous research has identified usual gait speed as a major predictor of stair-climbing performance among older adults [31], supporting stair-climbing performance as a reasonable proxy for mobility-related slowness. In addition, its validity as a functional mobility assessment tool has been supported through significant correlations with the mobility domain of the EQ-5D-5L [23]. Accordingly, responses of “slightly difficult (2 points),” “very difficult (3 points),” or “unable to climb (4 points)” were classified as slowness [28]. Fifth, low physical activity was defined as engaging in less than 150 min per week of moderate-intensity physical activity, less than 75 min per week of vigorous-intensity physical activity, or an equivalent combination of activities falling below 150 min per week of moderate-intensity equivalent, based on the International Physical Activity Questionnaire (IPAQ) and the WHO physical activity guidelines [32].

### 2.7. Control Variables

Age, marital status, educational level, household income, alcohol drinking, smoking status, hypertension, and diabetes were selected as control variables, considering factors known to be associated with SCD, depressive symptoms, and frailty. The importance of these variables has been confirmed in numerous prior studies conducted in South Korea and internationally [13,14,24,33]. Age was categorized into 65–74 years and 75 years and older. Marital status was classified as cohabiting (with a spouse) and non-cohabiting (divorced, bereaved, separated, or unmarried). Educational level was divided into three categories: elementary school or below, middle school, and high school or above. Household income was divided into quartiles based on monthly income, with the first quartile representing the lowest income group. Alcohol consumption was defined as non-drinking for those who had never consumed alcohol or had consumed less than one drink per month in the past year, and as drinking for those who had consumed one or more drinks per month in the past year. Smoking status was classified as current smoker or non-smoker (former smoker or never smoker). Hypertension was classified as “yes” if systolic blood pressure was 140 mmHg or higher, diastolic blood pressure was 90 mmHg or higher, or the participant was currently taking antihypertensive medication, and as “no” otherwise. Diabetes was classified as “yes” if fasting blood glucose was 126 mg/dL or higher, the participant had received a physician’s diagnosis, was taking hypoglycemic agents or using insulin injections, or had a glycated hemoglobin level of 6.5% or higher, and as “no” otherwise.

### 2.8. Ethical Considerations

The raw data and user guidelines on the KNHANES website were reviewed before submitting a data analysis request. The data were provided with approval from the Korea Disease Control and Prevention Agency, and all data were anonymized to prevent individual identification. As this study is a secondary data analysis utilizing publicly available data, the requirement for informed consent was waived, and the study was granted an exemption from review by the Institutional Review Board of Gyeongsang National University (IRB No: GIRB-D25-NX-0063).

### 2.9. Data Analysis

Complex sample analyses incorporating strata, clusters, and weights were conducted using IBM SPSS version 28.0 (IBM Corp., Armonk, NY, USA) to reflect the complex sampling design of KNHANES. Standard errors were estimated using the Taylor series linearization method, which accounts for intra-cluster correlation and design effects to ensure valid statistical inference [34].

Multicollinearity among the independent variables was assessed using variance inflation factors (VIF) and tolerance values. The VIF values ranged from 1.02 to 1.46, and the tolerance values ranged from 0.69 to 0.98, indicating no multicollinearity concerns.

The characteristics of the participants and the main study variables were analyzed using complex sample frequency analyses and descriptive statistics. Differences between men and women were assessed using Rao–Scott χ^2^ tests for categorical variables and complex sample general linear models for continuous variables. The same procedures were used to compare the characteristics of participants excluded due to missing data (*n* = 432) with those included in the final analytic sample (*n* = 1404).

To examine moderating effects, complex sample general linear models were employed, and moderation was assessed by incorporating interaction terms between SCD and each FFP component. A hierarchical approach was applied. In Step 1, a baseline model was fitted to examine the association between SCD and depressive symptoms while controlling for covariates. In Step 2, a main effects model was constructed by adding all five FFP components as main effects to the Step 1 model. In Step 3, the interaction term between SCD and each FFP component was tested individually in separate models to evaluate moderating effects; each model included SCD, covariates, all five FFP component main effects, and a single interaction term. The Step 3 interaction analyses were exploratory and intended to identify candidate moderators for the final parsimonious model (Step 4), rather than to test pre-specified hypotheses. In Step 4, a final parsimonious model was constructed by retaining SCD, covariates, and all five FFP component main effects, while simultaneously including only the interaction terms that reached statistical significance in Step 3. This step was included to confirm the unique moderating effect of each significant interaction term while controlling for other significant moderators. The threshold for statistical significance was set at *p* < 0.05, and changes in the coefficient of determination (ΔR^2^) and the overall model Wald F statistic were reported as indicators of model fit.

For each moderator identified as significant in Step 3, simple slope analyses were additionally conducted to estimate the association between SCD and depressive symptoms separately for participants with and without the corresponding frailty component.

To formally test whether the moderating effect of each frailty component differed significantly by sex, additional complex sample general linear models were conducted using the combined-sex sample. Each model included SCD, sex, the corresponding frailty component, all two-way interactions among these variables, the three-way interaction term (SCD × frailty component × sex), and the same eight covariates.

For the interaction analyses, SCD was treated as a continuous variable, with higher scores indicating greater subjective cognitive decline. Each frailty component was coded as a binary indicator (0 = criterion not met, 1 = criterion met), with the absence of each frailty component (coded 0) serving as the reference category.

All models were adjusted for covariates, including age, marital status, education level, household income, alcohol consumption, smoking status, hypertension, and diabetes. To account for potential sex differences, sex-stratified analyses were conducted separately for men and women. R^2^ values represent unadjusted coefficients of determination derived from complex sample general linear models.

## 3. Results

### 3.1. Sample Characteristics

The final analytic sample comprised 1404 Korean adults aged 65 years and older, consisting of 643 men (45.6%) and 761 women (54.4%). The characteristics of participants excluded due to missing data (*n* = 432) were compared with those included in the final analytic sample (*n* = 1404), and the results are presented in Supplementary Table S1. Table 1 presents the sociodemographic and clinical characteristics of the study participants by sex. Significant sex differences were observed across numerous variables. Compared with men, women were more likely to be aged 75 years and older (41.4% vs. 34.3%, *p* = 0.018) and to be non-cohabiting (44.6% vs. 13.1%, *p* < 0.001).

Significant sex differences were observed in educational level; women had a higher proportion of elementary school education or below (53.8% vs. 31.5%, *p* < 0.001), whereas men had a higher proportion of high school education or above (48.8% vs. 25.3%). Additionally, significant sex differences were observed in household income distribution, with women more concentrated in the lowest income bracket (46.5% vs. 34.8%, *p* < 0.001).

Regarding health behavior variables, men showed significantly higher rates of alcohol consumption (55.5% vs. 17.5%, *p* < 0.001) and smoking (19.0% vs. 1.7%, *p* < 0.001) than women. For clinical conditions, the prevalence of diabetes was higher in men (30.1% vs. 22.6%, *p* = 0.003) than in women, whereas no significant sex difference was observed in hypertension (63.6% vs. 61.2%, *p* = 0.327).

Among frailty components, significant sex differences were observed in exhaustion and physical function. Women demonstrated higher rates of exhaustion (46.0% vs. 32.3%, *p* < 0.001) and slowness (74.2% vs. 57.9%, *p* < 0.001), and low physical activity (70.0% vs. 60.9%, *p* = 0.002) than men. However, unintentional weight loss and muscle weakness showed no significant sex differences.

Depressive symptoms scores were significantly higher in women than in men (M = 1.56, SE = 0.02 vs. M = 1.40, SE = 0.03, *p* < 0.001), whereas no significant sex difference was observed in SCD scores (M = 1.81, SE = 0.02 vs. M = 1.76, SE = 0.02, *p* = 0.145).

### 3.2. Sex-Stratified Associations Between SCD and Depressive Symptoms

As shown in Table 2 (Step 1), after adjusting for relevant covariates, a significant positive association was observed between SCD and depressive symptoms in both men (B = 0.31, *p* < 0.001) and women (B = 0.42, *p* < 0.001).

Among the covariates, age (B = −0.16, *p* = 0.003), marital status (B = 0.24, *p* = 0.005), alcohol consumption (B = −0.13, *p* = 0.005), and smoking (B = 0.16, *p* = 0.012) were significantly associated with depressive symptoms in men, whereas only diabetes (B = −0.15, *p* = 0.008) showed a significant association in women.

### 3.3. Sex-Stratified Moderating Effects of Frailty Components on the Association Between SCD and Depressive Symptoms

Table 3 and Table 4 present the results of the hierarchical, complex-samples moderated regression analyses. To examine moderating effects, interaction terms between SCD and FFP components were tested within each sex.

In Step 2, a significant positive association between SCD and depressive symptoms was observed in both men (B = 0.26, *p* < 0.001) and women (B = 0.34, *p* < 0.001). Among the main effects of frailty, exhaustion (B = 0.28, *p* < 0.001) and slowness (B = 0.12, *p* = 0.012) were significantly associated with depressive symptoms in men. Similarly, exhaustion (B = 0.22, *p* < 0.001) and slowness (B = 0.17, *p* = 0.001) were significantly associated with depressive symptoms in women. The Step 2 models accounted for 24% and 20% of the variance in depressive symptoms for men (Wald F = 8.09, *p* < 0.001) and women (Wald F = 12.28, *p* < 0.001), respectively.

To examine each frailty component as a potential moderator while accounting for potential overlap among components, each SCD × frailty interaction term was tested in a separate model in Step 3. Among men, significant interactions were identified for unintentional weight loss (B = 0.44, *p* = 0.012), exhaustion (B = 0.19, *p* = 0.032), and slowness (B = 0.14, *p* = 0.049). Among women, significant interactions were observed for muscle weakness (B = −0.25, *p* = 0.002), slowness (B = 0.18, *p* = 0.019), and unintentional weight loss (B = −0.30, *p* = 0.029). Subgroup sizes and the complete Step 3 interaction results are presented in Supplementary Tables S2 and S4, respectively.

A final parsimonious model (Step 4) was constructed, retaining only the interaction terms that reached significance in Step 3. Among men, the interactions of SCD with unintentional weight loss (B = 0.43, *p* = 0.009) and exhaustion (B = 0.18, *p* = 0.037) remained statistically significant, whereas the interaction with slowness showed no statistical significance (*p* = 0.234). Among women, muscle weakness (B = −0.26, *p* = 0.002), slowness (B = 0.18, *p* = 0.020), and unintentional weight loss (B = −0.34, *p* = 0.022) were identified as significant interaction terms.

**Figure 1:**
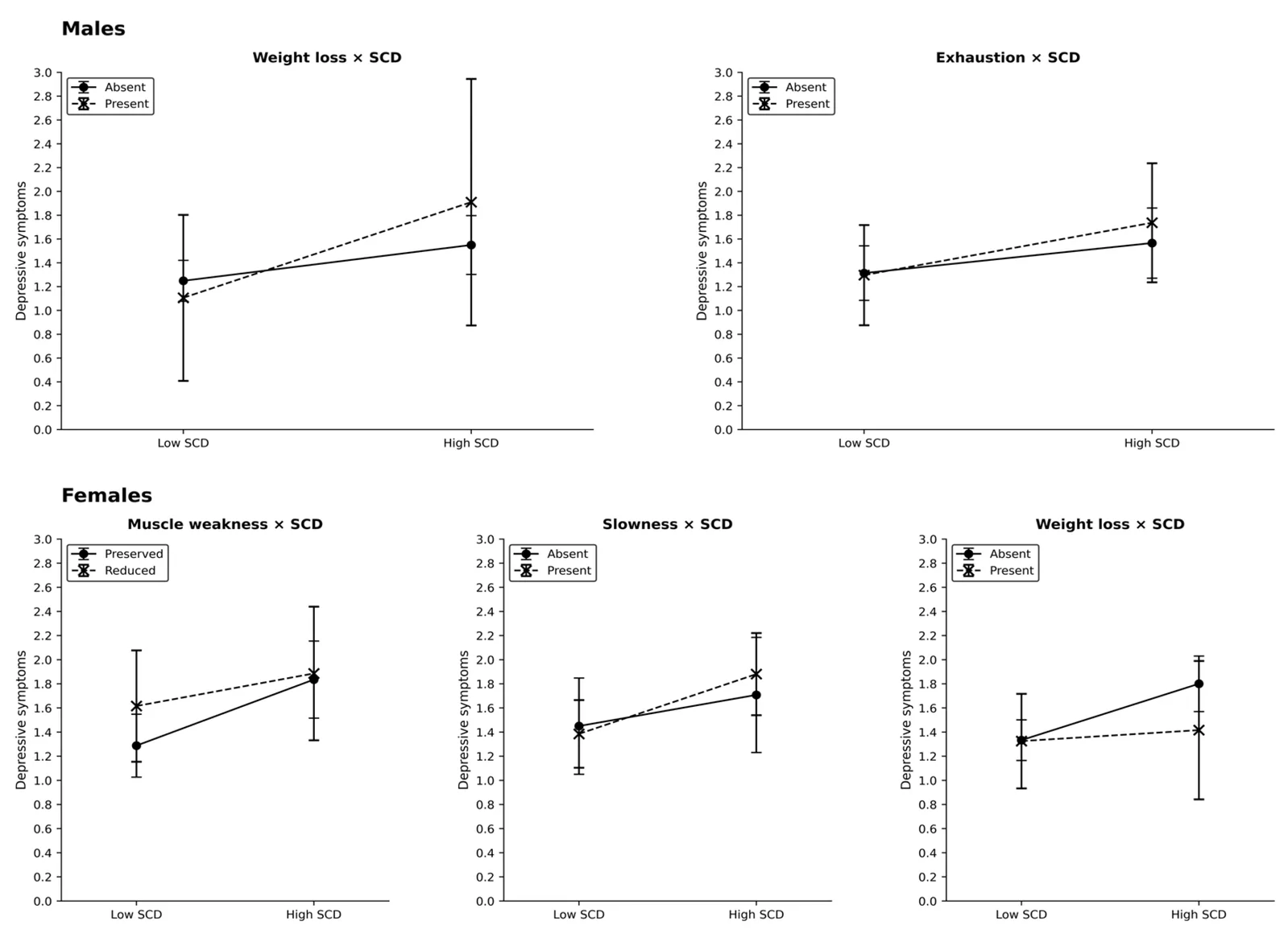
Moderation effects of frailty components on the association between SCD and depressive symptoms by sex. Note. SCD = subjective cognitive decline. Estimated scores and 95% CIs were derived from subgroup-specific regression models (Supplementary Table S3). Only the significant interactions identified in the Step 4 models are shown. Low SCD = 1.20 and High SCD = 2.38 represent one standard deviation below and above the unweighted analytic sample mean, respectively (M = 1.79, SD = 0.59). Slowness was assessed by difficulty in stair climbing. For unintentional weight loss, estimates are unadjusted because the covariate-adjusted model produced a near-singular design-based covariance matrix; all other estimates are covariate-adjusted.

Specifically, among men, the association between SCD and depressive symptoms was more pronounced in those with unintentional weight loss and exhaustion. Among women, the association between SCD and depressive symptoms was stronger in those without muscle weakness and without unintentional weight loss, as well as those with slowness (Figure 1). Detailed simple slope estimates are presented in Supplementary Table S3. The final models accounted for 26% (ΔR^2^ = 0.020, Wald F = 7.56, *p* < 0.001) and 21% (ΔR^2^ = 0.017, Wald F = 11.09, *p* < 0.001) of the variance in depressive symptoms for men and women, respectively.

## 4. Discussion

In this study, we examined whether individual components of FFP moderate the association between SCD and depressive symptoms among community-dwelling older adults, and whether these moderating effects differ between men and women. The positive association between SCD and depressive symptoms was observed in both men and women, consistent with prior studies [8,9,24]. Older adults with SCD often experience worry and anxiety related to memory loss, and a systematic review has reported that subjective cognitive change is associated with depressive symptoms [35]. Furthermore, the association between SCD and depressive symptoms has been reported to vary according to the level of frailty [13]. Nascimento et al. [13] reported that higher levels of frailty were associated with a stronger relationship between cognitive function and depressive symptoms. Our study findings are partially consistent with those of Nascimento et al. in that frailty may moderate the association between cognition-related variables and depressive symptoms. However, unlike prior research that analyzed the moderating role of frailty as a unified construct, we examined the moderating effect of each FFP component separately within sex-stratified samples. The analyses revealed that unintentional weight loss and exhaustion were significant moderators in men, whereas slowness, muscle weakness, and unintentional weight loss were significant moderators in women. To the best of our knowledge, no prior studies have directly examined the moderating role of individual FFP components in the association between SCD and depressive symptoms, thereby limiting direct comparisons with the existing literature. Nevertheless, our findings suggest that the pattern of FFP components moderating the association between SCD and depressive symptoms may differ between older men and older women, depending on the specific FFP components involved.

Among older men, the association between SCD and depressive symptoms was significantly stronger in those with unintentional weight loss and in those with exhaustion. Unintentional weight loss has been reported as a key indicator of physical decline in older adults [36], and its association with depressive symptoms has also been documented in prior research [37]. Exhaustion, a frailty component characterized by chronic low energy and fatigue [11], is relevant in this context, as fatigue has been found to be positively associated with SCD [38] and has been longitudinally associated with depressive symptoms over five years [39]. Individuals tend to use physical symptoms as cues for evaluating their health status [40], and older men in particular have been reported to convert physical symptoms into emotional distress [41]. Given these prior findings, the stronger association between SCD and depressive symptoms observed among older men with unintentional weight loss or exhaustion in the present study may be interpreted accordingly. However, as studies directly examining the mechanisms underlying this pattern are scarce, further research is warranted.

Notably, the pattern of FFP components moderating the association between SCD and depressive symptoms differed in direction among older women depending on the component observed. Concerning slowness (assessed using difficulty in stair climbing as a proxy), the association between SCD and depressive symptoms was stronger in those with decreased physical function, a pattern similar to that observed among older men with unintentional weight loss or exhaustion. In contrast, a paradoxical pattern was observed for muscle weakness and unintentional weight loss, where the association was stronger among those who did not meet these frailty criteria. Women have been reported to experience SCD more severely than men [42], and greater SCD severity has been associated with poorer emotional health, including depressive symptoms in women [43,44]. Considering these findings, the stronger association between SCD and depressive symptoms observed among physically healthy older women in the present study may be understood within this context. However, studies directly investigating the mechanisms underlying this mixed pattern are limited; hence, further research is needed.

The present study offers theoretical and clinical significance by evaluating frailty at the level of individual components rather than as a single unified construct, and by suggesting that the moderating roles of individual frailty components may differ between men and women. Previous studies have typically analyzed the moderating role of frailty as a unified construct; however, the present study examined the moderating role of each of the five FFP components individually, thereby identifying specific aspects of frailty associated with the SCD–depressive symptoms relationship at the component level. Testing each interaction term in a separate model revealed that unintentional weight loss and exhaustion were significant moderators in men, whereas slowness, muscle weakness, and unintentional weight loss were significant moderators in women. Furthermore, an additional analysis directly testing sex differences in moderating effects identified a statistically significant three-way interaction only for unintentional weight loss. This finding provides statistical support for the possibility that the moderating effect of unintentional weight loss may differ by sex and highlights the importance of considering sex when evaluating the moderating roles of individual frailty components. However, given the small subgroup size, this estimate should be interpreted with caution. The use of the KNHANES 2023 data, a nationally representative sample, along with weighted analyses reflecting the complex sampling design, strengthens the generalizability of our results. Moreover, treating SCD and depressive symptoms as continuous variables enabled a more nuanced representation of individual differences and increased statistical power, thereby constituting an additional methodological strength.

This study has several limitations. First, the cross-sectional design precludes determination of the directionality or temporal sequence of relationships among the variables, and the possibility of reverse causation cannot be ruled out. Second, several measurement-related limitations should be noted. Both SCD and depressive symptoms were assessed using single self-report items from the HINT-8, which may not have fully captured these multidimensional constructs. In addition, because SCD (memory item), depressive symptoms (depression item), and exhaustion (vitality item) were assessed using different items from the same HINT-8 instrument, the possibility of common-method bias [45] cannot be excluded. Therefore, the present findings should be interpreted with caution, and future studies should validate these findings using standardized, validated multi-item instruments. Third, the operational definitions of the frailty components varied in their correspondence to the original FFP criteria. Unintentional weight loss, muscle weakness, and low physical activity were operationalized using epidemiological and clinical criteria broadly consistent with the original FFP. In contrast, exhaustion and slowness were assessed using single self-reported HINT-8 items as proxy measures because standardized performance-based measures were unavailable in KNHANES. Accordingly, findings regarding exhaustion and slowness should be interpreted in light of these measurement limitations. Fourth, participants excluded because of missing frailty-related data (*n* = 432) were more likely to be older, women, without a spouse, and to have lower income and educational attainment than those included in the analysis (Supplementary Table S1). This suggests the possibility of selection bias, and because older adults with poorer physical function may have been disproportionately excluded, the analytic sample may represent a relatively healthier subgroup than the general older population. Fifth, because each of the five frailty components was tested separately in an exploratory analysis, the possibility of Type I error due to multiple testing cannot be excluded. Indeed, a statistically significant sex difference in the moderating effect was confirmed only for unintentional weight loss in the three-way interaction analysis (Supplementary Table S4), whereas the apparent sex-specific patterns observed for the other frailty components were not statistically supported. Therefore, these findings should be interpreted as exploratory, and replication in independent populations is warranted.

Despite these limitations, the present findings offer several implications for future research and clinical practice. Longitudinal studies are needed to clarify the directionality and temporal sequence of the associations observed in this study. In addition, replication using standardized and validated measures of cognition, depressive symptoms, and frailty, including multi-item scales for cognition and depressive symptoms, and performance-based frailty assessments incorporating objective gait speed and handgrip strength, is needed to confirm the present findings. In particular, because the supplementary analysis conducted to examine sex differences (Supplementary Table S4) confirmed a statistically significant sex difference only for unintentional weight loss, further research is warranted to elucidate the biological and behavioral mechanisms underlying the observed sex difference in unintentional weight loss. Clinically, assessments of depressive symptoms in older adults with SCD should consider individual frailty components in addition to overall frailty status. In this study, unintentional weight loss and exhaustion in men, and slowness, muscle weakness, and unintentional weight loss in women, showed moderating effects on the association between SCD and depressive symptoms. However, the clinical significance of these findings and the reproducibility of the observed sex differences need to be confirmed through future longitudinal studies and replication in independent populations.

## 5. Conclusions

A significant positive association between SCD and depressive symptoms was observed in both older men and women. Among the individual FFP components, unintentional weight loss and exhaustion in older men, and slowness, muscle weakness, and unintentional weight loss in older women, showed significant moderating effects on the association between SCD and depressive symptoms. These findings support the value of examining frailty at the level of individual components and provide additional insight into the relationship between SCD and depressive symptoms in older adults. However, because this study was based on a cross-sectional design, these findings require confirmation through longitudinal studies and replication in independent populations.

## Figures and Tables

**Table 1:** Sociodemographic and main variable characteristics of participants by sex (n = 1404).

Variables	Total (*n* = 1404)	Men (*n* = 643, 45.6%)	Women (*n* = 761, 54.4%)	F/χ^2^ (*p*)
	%(SE)/M(SE)	%(SE)/M(SE)	%(SE)/M(SE)	
Age				
65–74	61.8 (1.6)	65.7 (2.1)	58.6 (2.2)	5.72 (0.018)
≥75	38.2 (1.6)	34.3 (2.1)	41.4 (2.2)	
Marital status				
Not cohabiting	30.2 (1.6)	13.1 (1.6)	44.6 (2.0)	146.63(<0.001)
Cohabiting	69.8 (1.6)	86.9 (1.6)	55.4 (2.0)	
Education level				
≤Elementary	43.6 (1.8)	31.5 (2.0)	53.8 (2.3)	50.16 (<0.001)
Middle school	20.3 (1.2)	19.7 (1.6)	20.8 (1.6)	
≥High school	36.1 (1.9)	48.8 (2.3)	25.3 (2.1)	
Household income				
Lowest	41.1 (2.0)	34.8 (2.3)	46.5 (2.3)	12.57 (<0.001)
Lower middle	29.0 (1.5)	29.2 (2.0)	28.8 (1.6)	
Upper middle	19.3 (1.4)	22.0 (1.9)	17.0 (1.5)	
Highest	10.6 (1.1)	14.0 (1.8)	7.7 (1.1)	
Alcohol consumption				
No	65.2 (1.2)	44.5 (2.3)	82.5 (1.4)	166.22 (<0.001)
Yes	34.8 (1.2)	55.5 (2.3)	17.5 (1.4)	
Smoking status				
No	90.4 (0.8)	81.0 (1.7)	98.3 (0.4)	126.76 (<0.001)
Yes	9.6 (0.8)	19.0 (1.7)	1.7 (0.4)	
Hypertension				
No	37.7 (1.4)	36.4(1.8)	38.8 (1.9)	0.97 (0.327)
Yes	62.3 (1.4)	63.6(1.8)	61.2 (1.9)	
Diabetes				
No	74.0 (1.4)	69.9 (2.0)	77.4 (1.7)	9.26 (0.003)
Yes	26.0 (1.4)	30.1 (2.0)	22.6 (1.7)	
Unintentional weight loss				
No	94.5 (0.6)	95.0 (0.9)	94.1 (0.9)	0.57 (0.451)
Yes	5.5 (0.6)	5.0 (0.9)	5.9 (0.9)	
Exhaustion				
No	60.2 (1.3)	67.7 (2.0)	54.0 (1.8)	24.10 (<0.001)
Yes	39.8 (1.3)	32.3 (2.0)	46.0 (1.8)	
Weakness				
No	80.6 (1.7)	81.0 (2.3)	80.3 (2.0)	0.07 (0.794)
Yes	19.4 (1.7)	19.0 (2.3)	19.7 (2.0)	
Slowness				
No	33.2 (1.4)	42.1 (2.0)	25.8 (1.7)	45.32 (<0.001)
Yes	66.8 (1.4)	57.9 (2.0)	74.2 (1.7)	
Low physical activity				
No	34.1 (1.5)	39.1 (2.1)	30.0 (2.0)	9.98 (0.002)
Yes	65.9 (1.5)	60.9 (2.1)	70.0 (2.0)	
Continuous variables				
SCD	1.78 (0.02)	1.76 (0.02)	1.81 (0.02)	−1.47 (0.145)
depressive symptoms	1.49 (0.02)	1.40 (0.03)	1.56 (0.02)	−4.97 (<0.001)

% and SE: weighted estimates using the complex sample design; M: mean; F and χ^2^ statistics and *p*-values are from complex sample general linear models or Rao–Scott adjusted chi-square tests; SCD: subjective cognitive decline. In this analysis, the ‘Slowness’ criterion was operationalized as ‘Difficulty in stair climbing.’.

**Table 2:** Sex-stratified adjusted regression analysis of the association between subjective cognitive decline and depressive symptoms: Step 1 Baseline Model (*n* = 1404).

Variables	Classification	Males				Females			
		B	SE	95% CI	*p*	B	SE	95% CI	*p*
Age (ref: 65–74)	≥75	−0.16	0.05	[−0.26, −0.05]	0.003	0.05	0.06	[−0.07, 0.17]	0.395
Marital status (ref: Cohabiting)	Not cohabiting	0.24	0.08	[0.07, 0.40]	0.005	−0.04	0.05	[−0.13, 0.05]	0.395
Education level (ref: ≥High school)	≤Elementary	−0.11	0.06	[−0.22, 0.00]	0.054	−0.02	0.06	[−0.14, 0.10]	0.720
	Middle school	−0.03	0.07	[−0.17, 0.10]	0.617	0.14	0.07	[−0.01, 0.28]	0.067
Household income (ref: Highest)	Lowest	0.09	0.08	[−0.06, 0.23]	0.265	−0.06	0.09	[−0.25, 0.12]	0.486
	Lower middle	−0.04	0.07	[−0.18, 0.11]	0.611	−0.05	0.09	[−0.22, 0.12]	0.558
	Upper middle	−0.04	0.08	[−0.20, 0.12]	0.625	0.09	0.10	[−0.11, 0.30]	0.360
Alcohol drinking (ref: No)	Yes	−0.13	0.05	[−0.22, −0.04]	0.005	−0.03	0.06	[−0.15, 0.08]	0.563
Smoking status (ref: No)	Yes	0.16	0.06	[0.04, 0.28]	0.012	0.28	0.32	[−0.35, 0.91]	0.385
Hypertension (ref: No)	Yes	0.05	0.05	[−0.05, 0.15]	0.335	−0.03	0.06	[−0.14, 0.09]	0.658
Diabetes (ref: No)	Yes	0.05	0.05	[−0.05, 0.14]	0.319	−0.15	0.06	[−0.26, −0.04]	0.008
SCD ^†^		0.31	0.04	[0.23, 0.38]	<0.001	0.42	0.04	[0.34, 0.49]	<0.001
Overall model Wald F		9.61 ***				10.95 ***			
R^2^		0.171				0.150			

Results represent the Step 1 baseline model examining the association between subjective cognitive decline and depressive symptoms, adjusted for covariates only. All analyses were conducted using a complex sample design incorporating sampling weights, stratification, and clustering. ^†^ Higher scores indicate greater levels of the variable. SCD = subjective cognitive decline; SE = standard error; CI = confidence interval. *** *p* < 0.001.

**Table 3:** Moderating effect of frailty on the association between subjective cognitive decline and depressive symptoms in men: Results from complex sample regression (*n* = 643).

Variables	Step 2				Step 4			
	B	SE	95% CI	*p*	B	SE	95% CI	*p*
Frailty main effects								
SCD ^†^	0.26	0.04	[0.18, 0.33]	<0.001	0.12	0.05	[0.02, 0.23]	0.023
Unintentional weight loss [ref = no]	0.13	0.11	[−0.09, 0.36]	0.241	−0.65	0.25	[−1.14, −0.17]	0.009
Exhaustion [ref = no]	0.28	0.06	[0.16, 0.40]	<0.001	−0.05	0.15	[−0.33, 0.24]	0.757
Weakness [ref = no]	−0.05	0.06	[−0.16, 0.06]	0.386	−0.05	0.06	[−0.16, 0.06]	0.377
Slowness [ref = no]	0.12	0.05	[0.03, 0.22]	0.012	−0.02	0.12	[−0.26, 0.22]	0.875
Low physical activity [ref = no]	−0.06	0.05	[−0.15, 0.04]	0.231	−0.06	0.05	[−0.16, 0.03]	0.186
Interaction terms								
SCD × Unintentional weight loss	—	—	—	—	0.43	0.16	[0.11, 0.76]	0.009
SCD × Exhaustion	—	—	—	—	0.18	0.09	[0.01, 0.34]	0.037
SCD × Slowness	—	—	—	—	0.09	0.07	[−0.06, 0.23]	0.234
SCD × Weakness	—	—	—	—	—	—	—	—
SCD × Low physical activity	—	—	—	—	—	—	—	—
Overall model Wald F	8.09 ***				7.56 ***			
R^2^	0.240				0.260			
ΔR^2^ (from Step 2)	—				0.020			

Step 2: Main effects model including SCD, all five frailty components, and covariates. Step 4: Final parsimonious model including SCD, all five frailty components, covariates, and only statistically significant interaction terms identified in Step 3 (*p* < 0.05). All models were adjusted for age, marital status, educational level, household income, alcohol drinking, smoking status, hypertension, and diabetes. Reference category (0) = criterion absent; 1 = criterion present. ^†^ Higher scores indicate greater levels of the variable. SCD = subjective cognitive decline; SE = standard error; CI = confidence interval. All analyses were conducted using a complex sample design incorporating sampling weights, stratification, and clustering. *** *p* < 0.001.

**Table 4:** Moderating effect of frailty on the association between subjective cognitive decline and depressive symptoms in women: Results from complex sample regression (*n* = 761).

Variables	Step 2				Step 4			
	B	SE	95% CI	*p*	B	SE	95% CI	*p*
Frailty main effects								
SCD ^†^	0.34	0.04	[0.26, 0.42]	<0.001	0.30	0.07	[0.16, 0.43]	<0.001
Unintentional weight loss [ref = no]	−0.14	0.09	[−0.31, 0.03]	0.103	0.48	0.25	[−0.01, 0.97]	0.056
Exhaustion [ref = no]	0.22	0.06	[0.11, 0.33]	<0.001	0.23	0.06	[0.12, 0.34]	<0.001
Weakness [ref = no]	−0.05	0.06	[−0.16, 0.06]	0.398	0.44	0.16	[0.13, 0.75]	0.005
Slowness [ref = no]	0.17	0.05	[0.07, 0.26]	0.001	−0.14	0.12	[−0.38, 0.10]	0.239
Low physical activity [ref = no]	−0.01	0.06	[−0.12, 0.09]	0.794	−0.01	0.05	[−0.11, 0.10]	0.898
Interaction terms								
SCD × Muscle weakness	—	—	—	—	−0.26	0.08	[−0.42, −0.10]	0.002
SCD × Slowness	—	—	—	—	0.18	0.08	[0.03, 0.33]	0.020
SCD × Unintentional weight loss	—	—	—	—	−0.34	0.15	[−0.63, −0.05]	0.022
SCD × Exhaustion	—	—	—	—	—	—	—	—
SCD × Low physical activity	—	—	—	—	—	—	—	—
Overall model Wald F	12.28 ***				11.09 ***			
R^2^	0.196				0.213			
ΔR^2^ (from Step 2)	—				0.017			

Step 2: Main effects model including SCD, all five frailty components, and covariates. Step 4: Final parsimonious model including SCD, all five frailty components, covariates, and only statistically significant interaction terms identified in Step 3 (*p* < 0.05). All models were adjusted for age, marital status, educational level, household income, alcohol drinking, smoking status, hypertension, and diabetes. Reference category (0) = criterion absent; 1 = criterion present. ^†^ Higher scores indicate greater levels of the variable. SCD = subjective cognitive decline; SE = standard error; CI = confidence interval. All analyses were conducted using a complex sample design incorporating sampling weights, stratification, and clustering. *** *p* < 0.001.

## Data Availability

The data presented in this study are from the Korea National Health and Nutrition Examination Survey (KNHANES) 2023 at https://knhanes.kdca.go.kr, operated by the Korea Disease Control and Prevention Agency.

## Supplementary Materials

The following supporting information can be downloaded at https://www.mdpi.com/article/10.3390/healthcare14162495/s1, Table S1: Comparison of sociodemographic and health-related characteristics between included and excluded participants; Table S2: Weighted prevalence and unweighted cell counts of modified Fried Frailty Phenotype components overall and by sex; Table S3: Simple slope analyses for the association between SCD and depressive symptoms, by significant frailty-component moderators and sex; Table S4: Sex-stratified SCD × frailty-component interaction models (Step 3) and formal three-way interaction tests.

## Abbreviations

The following abbreviations are used in this manuscript:

SCD	Subjective Cognitive Decline
FFP	Fried Frailty Phenotype
KNHANES	Korea National Health and Nutrition Examination Survey
VIF	Variance Inflation Factor
MCI	Mild Cognitive Impairment
HINT-8	Health-Related Quality of Life Instrument with 8 Items
IPAQ	International Physical Activity Questionnaire
