# Supplementary material for: Frailty Components as Moderators of the Subjective Cognitive Decline–Depressive Symptoms Association: A Sex-Stratified Population-Based Study
Source: Healthcare (Basel). 2026 Aug 11;14(16):2495. doi: 10.3390/healthcare14162495 (PMC13512530; doi:10.3390/healthcare14162495)
Supplement: Supplementary file 1 [file healthcare-14-02495-s001.zip › Supplementary_Table_S1.pdf]

Supplementary Table S1. Comparison of sociodemographic and health-related characteristics between included and excluded participants.

Variables	Total (n = 1,836)	Included (n = 1,404)	Excluded (n = 432)	F/χ² (p)
	%(SE)/M(SE)	*%(SE)/M(SE)*	*%(SE)/M(SE)*	
**Age**				
65–74	58.4 (1.5)	61.8 (1.6)	47.3 (3.1)	
≥75	41.6 (1.5)	38.2 (1.6)	52.7 (3.1)	18.11 (<0.001)
**Sex**				
Men	43.9 (0.9)	45.6 (1.0)	38.5 (2.2)	
Women	56.1 (0.9)	54.4 (1.0)	61.5 (2.2)	7.72 (0.006)
**Marital status**				
Not cohabiting	33.1 (1.5)	30.2 (1.5)	42.4 (2.7)	
Cohabiting	66.9 (1.5)	69.8 (1.5)	57.6 (2.7)	20.23 (<0.001)
**Household income**				
Lowest	45.2 (1.9)	41.2 (2.0)	58.5 (2.9)	
Lower middle	27.8 (1.2)	29.0 (1.5)	24.0 (2.1)	
Upper middle	17.3 (1.2)	19.3 (1.4)	10.6 (1.6)	
Highest	9.7 (1.0)	10.6 (1.1)	6.9 (1.6)	11.62 (<0.001)
**Education level**				
≤Elementary	45.6 (1.6)	43.6 (1.8)	53.9 (3.4)	
Middle school	20.1 (1.0)	20.3 (1.2)	19.0 (2.4)	
≥High school	34.3 (1.7)	36.1 (1.9)	27.2 (2.8)	4.83 (0.009)
**Self-rated health**				
Poor	26.5 (1.2)	25.5 (1.2)	32.7 (4.6)	
Fair	46.9 (1.5)	47.1 (1.5)	46.2 (3.8)	
Good	26.5 (1.3)	27.4 (1.5)	21.1 (2.9)	2.36 (0.106)
**Sleep duration**				
<6h	23.7 (1.1)	22.6 (1.2)	27.9 (2.8)	
6–8h	68.3 (1.2)	69.5 (1.3)	63.2 (3.2)	
>8h	8.1 (0.7)	7.9 (0.7)	8.8 (1.9)	1.99 (0.139)
**Stress**				
No	90.1 (0.8)	90.4 (0.8)	89.0 (1.8)	
Yes	9.9 (0.8)	9.6 (0.8)	11.0 (1.8)	0.55 (0.461)
**Continuous variables**				
SCD	1.79 (0.01)	1.783 (0.016)	1.817 (0.038)	-0.80 (0.424)

Variables	Total (n = 1,836)	Included (n = 1,404)	Excluded (n = 432)	F/χ² (p)
Depressive symptoms	1.50 (0.02)	1.487 (0.019)	1.540 (0.050)	-0.98 (0.328)

Values are weighted % (SE) for categorical variables and weighted mean (SE) for continuous variables, accounting for the KNHANES complex sampling design. F/χ^2 values are Rao–Scott-corrected statistics from tests of independence or complex-samples general linear models, respectively. Fried Frailty Phenotype components are not shown because these variables were, by definition, missing among excluded participants. Sample sizes for individual variables may not sum to totals due to item-level missing data.
