# Supplementary material for: Frailty Components as Moderators of the Subjective Cognitive Decline–Depressive Symptoms Association: A Sex-Stratified Population-Based Study
Source: Healthcare (Basel). 2026 Aug 11;14(16):2495. doi: 10.3390/healthcare14162495 (PMC13512530; doi:10.3390/healthcare14162495)
Supplement: Supplementary file 1 [file healthcare-14-02495-s001.zip › Supplementary_Table_S2.pdf]

Supplementary Table S2. Weighted prevalence and unweighted cell counts of modified Fried Frailty Phenotype components overall and by sex.

**Frailty component**	**Men (n = 643)**		**Women (n = 761)**		**F (*p*)**
	n	*%(SE)*	*n*	*%(SE)*	
**Unintentional weight loss**					
Yes	33	5.0 (0.9)	47	5.9 (0.9)	
No	610	95.0 (0.9)	714	94.1 (0.9)	0.57 (0.451)
**Exhaustion**					
Yes	215	32.3 (2.0)	346	46.0 (1.8)	
No	428	67.7 (2.0)	415	54.0 (1.8)	24.10 (<0.001)
**Weakness**					
Yes	118	19.0 (2.3)	139	19.7 (2.0)	
No	525	81.0 (2.3)	622	80.3 (2.0)	0.07 (0.794)
**Slowness**					
Yes	377	57.9 (2.0)	565	74.2 (1.7)	
No	266	42.1 (2.0)	196	25.8 (1.7)	45.32 (<0.001)
**Low physical activity**					
Yes	403	60.9 (2.1)	535	70.0 (2.0)	
No	240	39.1 (2.1)	226	30.0 (2.0)	9.98 (0.002)

% (SE) columns show weighted percentages with standard errors; n columns show unweighted cell counts, calculated using complex-samples analysis accounting for the KNHANES stratification, clustering, and sampling weights. F values are Rao–Scott second-order corrected F statistics from tests of independence comparing men and women.
