# Supplementary material for: Frailty Components as Moderators of the Subjective Cognitive Decline–Depressive Symptoms Association: A Sex-Stratified Population-Based Study
Source: Healthcare (Basel). 2026 Aug 11;14(16):2495. doi: 10.3390/healthcare14162495 (PMC13512530; doi:10.3390/healthcare14162495)
Supplement: Supplementary file 1 [file healthcare-14-02495-s001.zip › Supplementary_Table_S3.pdf]

Supplementary Table S3. Simple slope analyses for the association between SCD and depressive symptoms, by significant frailty-component moderators and sex.

Sex	Moderator	Group	n	B	SE	95% CI	p
Men	Unintentional weight loss[a]	Present	33	0.681	0.190	[0.277, 1.086]	0.003
		Absent	610	0.255	0.044	[0.168, 0.342]	<0.001
Men	Exhaustion	Present	200	0.373	0.067	[0.239, 0.507]	<0.001
		Absent	400	0.214	0.046	[0.123, 0.306]	<0.001
Women	Weakness	Present	130	0.229	0.076	[0.076, 0.382]	0.004
		Absent	586	0.464	0.046	[0.373, 0.555]	<0.001
Women	Slowness	Present	535	0.420	0.048	[0.326, 0.515]	<0.001
		Absent	181	0.219	0.065	[0.090, 0.348]	0.001
Women	Unintentional weight loss[a]	Present	47	0.077	0.104	[-0.137, 0.291]	0.466
		Absent	714	0.396	0.039	[0.319, 0.474]	<0.001

Values represent simple slope estimates for the association between SCD and depressive symptoms (B, unstandardized regression coefficient) within each level of the significant frailty component moderator, estimated using complex samples general linear models accounting for the KNHANES stratification, clustering, and sampling weights. Only frailty components with statistically significant SCD × frailty component interactions in the final parsimonious model (Step 4) are presented. Present and Absent indicate the presence and absence of the corresponding frailty component, respectively. Unless otherwise noted, all estimates were adjusted for age, marital status, educational level, household income, alcohol drinking, smoking status, hypertension, and diabetes. Subgroup sizes for the adjusted models reflect missing covariate data.

[a] Unadjusted simple slope estimates are reported for the unintentional weight-loss interaction because the covariate-adjusted model produced a near-singular design-based covariance matrix in the weight-loss-present subgroup ($n < 50$).
