# Supplementary material for: Frailty Components as Moderators of the Subjective Cognitive Decline–Depressive Symptoms Association: A Sex-Stratified Population-Based Study
Source: Healthcare (Basel). 2026 Aug 11;14(16):2495. doi: 10.3390/healthcare14162495 (PMC13512530; doi:10.3390/healthcare14162495)
Supplement: Supplementary file 1 [file healthcare-14-02495-s001.zip › Supplementary_Table_S4.pdf]

Supplementary Table S4. Sex-stratified SCD × frailty-component interaction models (Step 3) and formal three-way interaction tests.

Frailty component	Men: SCD × component B (SE), 95% CI, p	Women: SCD × component B (SE), 95% CI, p	Sex × SCD × component[a] B (SE), 95% CI, p
Weakness	–0.091 (0.107) [–0.302, 0.120] p = .397	–0.250 (0.082) [–0.411, –0.089] p = .002	–0.181 (0.145) [–0.468, 0.105] p = .213
Slowness	0.141 (0.071) [0.001, 0.280] p = .049	0.182 (0.077) [0.030, 0.334] p = .019	0.036 (0.101) [–0.163, 0.235] p = .722
Unintentional weight loss	0.435 (0.172) [0.096, 0.774] p = .012	–0.302 (0.138) [–0.573, –0.031] p = .029	–0.777 (0.243) [–1.258, –0.297] p = .002
Low physical activity	–0.043 (0.079) [–0.200, 0.113] p = .586	0.099 (0.093) [–0.084, 0.282] p = .289	0.076 (0.115) [–0.151, 0.302] p = .511
Exhaustion	0.188 (0.087) [0.017, 0.360] p = .032	0.045 (0.091) [–0.135, 0.224] p = .626	–0.167 (0.129) [–0.421, 0.088] p = .198

Men and women columns present the SCD × frailty-component interaction term from sex-stratified complex-samples general linear models, adjusted for age, marital status, educational level, household income, alcohol drinking, smoking status, hypertension, and diabetes (Step 3). The right-hand column presents the corresponding three-way interaction (SCD × frailty component × sex) from combined-sex models. SCD = subjective cognitive decline; SE = standard error; CI = confidence interval.

[a] A significant three-way interaction indicates that the moderating effect differed significantly between men and women.
